# Long Term Follow‐Up After Transplantation in Propionic Acidemia: A Retrospective French Pediatric and Adult Cohort Study

**DOI:** 10.1002/jimd.70216

**Published:** 2026-06-25

**Authors:** Tristan Mekdade, Claire‐Marine Bérat, Manuel Schiff, Margaux Gaschignard, Juliette Bouchereau, Jean‐Baptiste Arnoux, Claire Francoz, Aude Servais, Myriam Dao, Vickie Lacroix, Julien Maquet, Apolline Imbard, Edouard Le Guillou, Clément Pontoizeau, Jean‐François Benoist, Mehdi Oualha, Marion Grimaud, Charles de Marcellus, Laurent Sabbah, Claire Mayer, Christophe Chardot, Carmen Capito, Diala Khraiche, Florence Lacaille, Pascale de Lonlay, Anaïs Brassier

**Affiliations:** ^1^ Reference Center of Inherited Metabolic Diseases, Necker Enfants Malades Hospital, APHP, Université Paris Cité Paris France; ^2^ Pediatric Metabolic Unit, Pediatrics, Woman‐Mother‐Child Department Lausanne University Hospital Lausanne Switzerland; ^3^ INSERM UMR _S1163, Institut Imagine, Université Paris‐Cité Paris France; ^4^ Hepatology Unit, Beaujon Hospital, APHP Paris France; ^5^ Nephrology Unit, Necker Enfants Malades Hospital, APHP, Université Paris Cité Paris France; ^6^ Pediatric Gastroenterology, Hepatology and Metabolic Disease Toulouse University Hospital Toulouse France; ^7^ Internal Medicine, Toulouse University Hospital Toulouse France; ^8^ Biochemistry Department Necker‐Enfants Malades University Hospital, Assistance Publique‐Hôpitaux de Paris (AP‐HP) Paris France; ^9^ Pediatric Intensive Care Unit, Necker Enfants Malades Hospital, APHP, Paris Cité University Paris France; ^10^ Adult Cardiology Unit, Necker Enfants Malades Hospital, APHP, Université Paris Cité Paris France; ^11^ Pediatric Gastroenterology‐Nutrition and Hepatology Units, Necker Enfants Malades Hospital, APHP, Université Paris Cité Paris France; ^12^ Pediatric Liver Surgery, Necker Enfants Malades Hospital, APHP, Université Paris Cité Paris France; ^13^ Pediatric Cardiology Unit, Necker Enfants Malades Hospital, APHP, Université Paris Cité Paris France; ^14^ INSERM‐1151, INEM Paris France

**Keywords:** cardiomyopathy, Leigh syndrome, liver transplantation, propionic acidemia, psychiatric manifestations

## Abstract

Propionic acidemia (PA) is a rare inherited metabolic disorder associated with recurrent metabolic decompensations and chronic multisystemic complications. Liver transplantation (LT) may improve metabolic stability, but its long‐term impact on organ involvement remains debated. We retrospectively studied 20 patients with PA transplanted between 1993 and 2024 in three French reference centers. Clinical, biochemical, and organ‐specific data were collected before and after transplantation. Eighteen isolated LT and two combined liver–kidney transplantations were performed. Median age at transplantation was 13.6 years; median follow‐up was 4.5 years. Indications included frequent metabolic decompensation (70%) and cardiomyopathy (35%). Four patients died perioperatively, three from cardiac causes; crude survival was 75%. Transplantation markedly improved metabolic stability and allowed significant dietary liberalization, with reduced need for enteral feeding. Cardiac involvement, present in 15 patients pre‐transplant, showed variable evolution: 10 improved initially, but three deteriorated later, and one required heart transplantation. New neurological manifestations occurred in eight patients, with acute episodes including CNI‐related encephalopathy. Psychiatric disorders progressed from 40% to 59% of patients, requiring treatment in seven. Renal impairment worsened post‐LT to 70%, with mean measured glomerular filtration rate declining from 72 to 60 mL/min/1.73 m^2^. LT improves metabolic and nutritional outcomes but does not consistently prevent chronic organ complications. In conclusion, early LT, before the onset of cardiac, neurologic, and psychiatric manifestations, should be considered on a case‐by‐case basis, as we could not demonstrate correlation between age at transplantation and organ involvement.

## 1 Introduction

Propionic acidemia (PA, OMIM#606054) is a rare inherited metabolic disorder caused by pathogenic variants in the *PCCA* or *PCCB* genes, which encode the two subunits of mitochondrial propionyl‐CoA carboxylase (PCC; EC6.4.1.3). This enzyme converts propionyl‐CoA to methylmalonyl‐CoA; the latter is metabolized into succinyl‐CoA which enters the tricarboxylic acid cycle. Disruption of this process leads to accumulation of propionyl‐CoA and related metabolites, resulting in acute, life‐threatening metabolic crises and chronic multisystemic complications with long‐term consequences.

Currently, there is no curative treatment. Management relies on a strict protein‐restricted diet, prevention of prolonged fasting through enteral nutrition, ongoing L‐carnitine supplementation, and emergency interventions during catabolic stress [1, 2]. Despite optimal management, patients develop multiple complications—mainly cardiac, neurological, psychiatric, and renal—that significantly impact the overall prognosis of the disease [1, 3].

The first liver transplantation (LT) for PA was performed at our institution in 1993 [4]. Over the years, the paradigm of liver transplantation in inborn errors of metabolism has shifted from a life‐saving option to a consolidated life‐improving enzyme replacement therapy [5]: LT was shown to improve metabolic stability, reduce the frequency of metabolic decompensation events (MDEs), and mitigate chronic complications [1, 6, 7, 8]. However, LT does not fully prevent long‐term organ damage, particularly cardiac involvement and psychiatric manifestations, which markedly affect patients' quality of life [9, 10]. Moreover, patients with PA are particularly vulnerable; immediate postoperative management is often complicated by their metabolic fragility [7, 11].

This retrospective study, conducted across French pediatric and adult references centers for metabolic diseases and liver transplantation provides detailed longitudinal data on pre‐transplantation status and short‐ and long‐term post‐transplantation outcomes.

## 2 Patients and Methods

We included all children and adults with PA followed at the Reference Center for Inherited Metabolic Diseases at Necker–Enfants Malades who underwent liver transplantation at Necker (children), Beaujon (adults), or Toulouse (adults) hospitals between 1993 and 2024. PA diagnosis was confirmed by biochemical analysis and/or molecular genetic testing.

This retrospective, observational, noninterventional cohort study collected clinical and laboratory data from patients' medical records. Collected data included epidemiological characteristics, initial diagnosis, genetic testing results, metabolic status, and organ‐related complications prior to procedure, postoperative complications, and follow‐up assessments on metabolic status and organ function after transplantation. Metabolic parameters comprised dietary management details, criteria for MDEs (defined as acute metabolic ketoacidosis with increased anion gap and/or hyperammonemia and/or hyperlactatemia), and urinary 3‐hydroxypropionate (3‐OH‐p) levels as a marker of chronic metabolic balance.

Cardiac involvement was defined by the presence of dilated or hypertrophic cardiomyopathy, confirmed by transthoracic echocardiography performed by a specialist cardiologist.

Dilated cardiomyopathy was defined as left ventricular (LV) dilatation with global or regional systolic dysfunction not solely explained by abnormal loading conditions [12, 13].

Hypertrophic cardiomyopathy was defined as increased LV wall thickness or mass not solely explained by abnormal loading conditions [12, 13].

Left ventricular ejection fraction (LVEF) was considered reduced if below 50%, in accordance with American College of Cardiology/American Heart Association guidelines [14].

Post‐transplant cardiac improvement was defined as an increase in LVEF and/or a reduction in cardiac medications.

Chronic kidney disease was assessed according to the KDIGO classification: glomerular filtration rate (GFR) below 89 mL/min/1.73 m^2^ with persistent moderate albuminuria, or GFR < 60 mL/min/1.73 m^2^ irrespective of albuminuria [15]. GFR was measured by inulin or iohexol clearance.

Any neurologic, neurosensory, or psychiatric symptoms were recorded and included in the assessment. Cognitive impairment was graded as mild, moderate, or severe based on the treating clinician's assessment recorded in the medical charts, in the absence of systematic neuropsychological testing.

Metabolic balance was partially assessed using mean urinary 3‐hydroxypropionate levels. Comparison of pre‐ and post‐transplant values was based on the mean of all available 3‐OH‐p measurements recorded in patients' charts. Mean urinary tiglyglycine, propionylglycine, and 2‐methylcitric acid were also collected, along with plasma branched‐chain amino acids and glycine measured before transplantation and at last follow‐up.

In accordance with French legislation, patients or their legal representatives were informed during routine consultations that their medical data might be used for research purposes; lack of opposition was systematically recorded. All data were anonymized and stored securely in a password‐protected database, in compliance with French and European data protection regulations.

Comparisons of pre‐ and post‐LT values were performed using the Wilcoxon signed‐rank test for nonparametric data or the paired *t*‐test for normally distributed data. Normality was assessed with the Shapiro–Wilk test before selecting the appropriate test. A *p* value < 0.05 was considered statistically significant. Adjustments for multiple comparisons were made using the Bonferroni method. Additionally, a linear mixed‐effects model was used to account for between‐subject variability and repeated measures. Missing data were handled using a complete‐case analysis approach. Denominators were adjusted to reflect only patients for whom data were available for each specific variable; no imputation was performed. Descriptive statistics were generated, and statistical analyses were performed using R statistical software, version 4.2.3 (2023‐06‐16)–“Beagle Scouts” Copyright (C) 2023.

## 3 Results

Patients' characteristics are summarized in Table 1. The cohort included 20 patients (10 male, 10 female). All patients were born after uneventful pregnancies and deliveries. First symptoms appeared in the neonatal period for 17 patients and between 1 and 10 months of age for the remaining 3. Seventeen patients presented with ketoacidosis and acute neurologic deterioration progressing to coma; of these, 7 required hemodiafiltration at diagnosis. P9, diagnosed late at 10 months of age, presented with hypotonia and cortical blindness but had no MDEs; she subsequently recovered. Nineteen patients needed enteral nutrition pre‐transplantation to optimize nutritional and metabolic status.

**TABLE 1:** Demographic data.

Patient	P1	P2	P3	P4	P5	P6	P7	P8	P9	P10
Sex	M	F	M	F	M	F	M	F	F	F
Genetic	PCCB, homozygosity c.990dupT (p.E331X)	PCCB, compound heterozygosity c.441delG (p.Q147QfsX+2) and c.1218_1231del14ins12 (p.Gly406GlyfsX15)	NA	NA	PCCB, homozygosity c.990dupT (p.E331X)	PCCA, compound heterozygosity c.1284+4A>T and c.1288C>T (p.R430X)	PCCA, homozygosity del ex23	PCCA, homozygosity c.1409T>G (p.Leu490Arg)	PCCB, compound heterozygosity c.772A>G (p.H258R) and c.1126C>T (p.R376C)	NA
Initial symptoms	Coma	Coma, acidosis	Coma	Coma	Coma	Coma	Coma	NA	Cortical blindness, hypotonia	Coma, acidosis
Age at diagnosis	NN	NN	NN	NN	NN	1 month	NN	NN	10 months	NN
HDF	Yes	No	No	No	Yes	Yes	No	Yes	NA	Yes
Tx indication	CM	MDEs	CM	MDEs	CM + MDEs	CM + MDEs	Cirrhosis + PHT	CM	CM	MDEs
Graft type	LT	LKT	LT	LT	LT	LT	LT	LT	LT	LT
Liver graft type	WL	WL	WL	WL	WL	WL	LL	WL	LL	WL
Age at Tx (years)	16	20	19.8	9	14	8	6.5	22	6	24
Follow‐up (years)	6.6	4.5	5.3	18^a^	8.3	11.6	0^a^	3.6	19	5.9

Patient	P11	P12	P13	P14	P15	P16	P17	P18	P19	P20
Sex	F	F	M	F	M	M	F	M	M	M
Genetic	PCCA, homozygosity c.284DelA (p.Asp95Valfs9*)	PCCB, compound heterozygosity c.1218_1231del14ins12 (p.Gly406GlyfsX15) and c.1535G>A (p.Arg512His)	PCCB, homozygosity c.439C>T (p.Q147X)	NA	NA	PCCA, coumpound heterozygositz c.1456C>T and c.2062T>C	NA	PCCA, homozygosity c.776T>C (p.Leu259Pro)	NA	PCCA, homozygosity del ex23
Initial symptoms	Coma	Coma, acidosis	Coma, acidosis	Coma, acidosis	Coma	Coma, acidosis	Coma	Coma	Coma, acidosis	Coma, acidosis
Age at diagnosis	NN	NN	3 months	NN	NN	NN	NN	NN	NN	NN
HDF	No	No	No	No	No	Yes	NA	NA	Yes	NA
Tx indication	MDEs	CM	MDEs	MDEs	MDEs	MDEs	MDEs	MDEs	MDEs	MDEs + RI
Graft type	LT	LT	LT	LT	LT	LT	LT	LT	LT	LKT
Liver graft type	LL	RL	LL	WL	WL	LL	WL	WL	WL	WL
Age at Tx (years)	5.25	13.2	11.2	23	13	2.7	18	19	7	28
Follow‐up (years)	2.8	0^a^	1.8	2.7	1	0^a^	2.5	6.4	1.8^a^	3.4

Abbreviations: CM, cardiomyopathie; HDF, hemodiafiltration; LKT, liver kidney transplant; LL, left liver; LT, liver transplant; MDEs, frequent metabolic decompensation events; NA, not available; NN, neonatal; PHT, portal hypertension; RI, renal insufficiency; RL, right liver; Tx, transplantation; WL, whole liver.

^a^ Died.

P4 and P19 underwent the first LT in 1993, P9 in 2006, and the remaining patients after 2010.

Indications for transplantation included frequent MDEs (*n* = 14; 70%) with subsequent impaired metabolic control and/or cardiomyopathy (*n* = 7; 35%). One patient (P7) underwent LT for biliary cirrhosis complicated by portal hypertension, attributed to PA in the absence of any other identified etiology, with hepatomegaly documented from age 1 year and progression to cirrhosis by age four. The median age at first transplantation was 13 years 7 months (range: 2 years 9 months to 28 years), of whom seven underwent transplantation before the age of 10 years. Median follow‐up after initial transplantation was 4.5 years (range: 1–19 years). At last follow‐up, median patient age was 24.6 years.

Eighteen patients received isolated liver transplants (LT); two patients (P2 and P20) received a combined liver‐kidney transplantation (LKT). P4 underwent kidney transplantation (KT) for kidney failure 15 years after LT for kidney failure, followed by a second LT 2 years later for chronic rejection. P9 received a second LT 5 years after the first, for biliary cirrhosis occurring in the context of chemotherapy for an EBV‐induced Burkitt lymphoma. P1 underwent heart transplantation (HT) 5 years after LT.

Five patients died at a median age of 8.8 years (range: 2.7–27 years), of whom four occurred in the peri‐transplant period (P4, P7, P12, P16). P4 died of multi‐organ failure after re‐transplantation. P7 died post‐LT from refractory myocardial stunning: left ventricular dysfunction without dilatation was documented on day 3, progressing to ventricular fibrillation at the time of ECMO removal on day 7. P12 died following a cascade of complications including sepsis secondary to infection of a hepatic necrosis focus with suprahepatic venous ischemia, subsequent acute respiratory distress syndrome, and fatal cardiac arrest. P16 died from refractory ventricular tachycardia requiring ECMO, with subsequent cardiorespiratory arrest and brain death. P19 died 1 year and 8 months after LT: the post‐transplant course was complicated by acute rejection on day 12 (treated), hepatic artery thrombosis detected at 7 months, a post‐transplant lymphoproliferative disorder attributed to cyclosporine (which resolved with treatment), and progressive biliary cirrhosis with chronic cholangitis, ultimately leading to graft failure.

**FIGURE 1:**
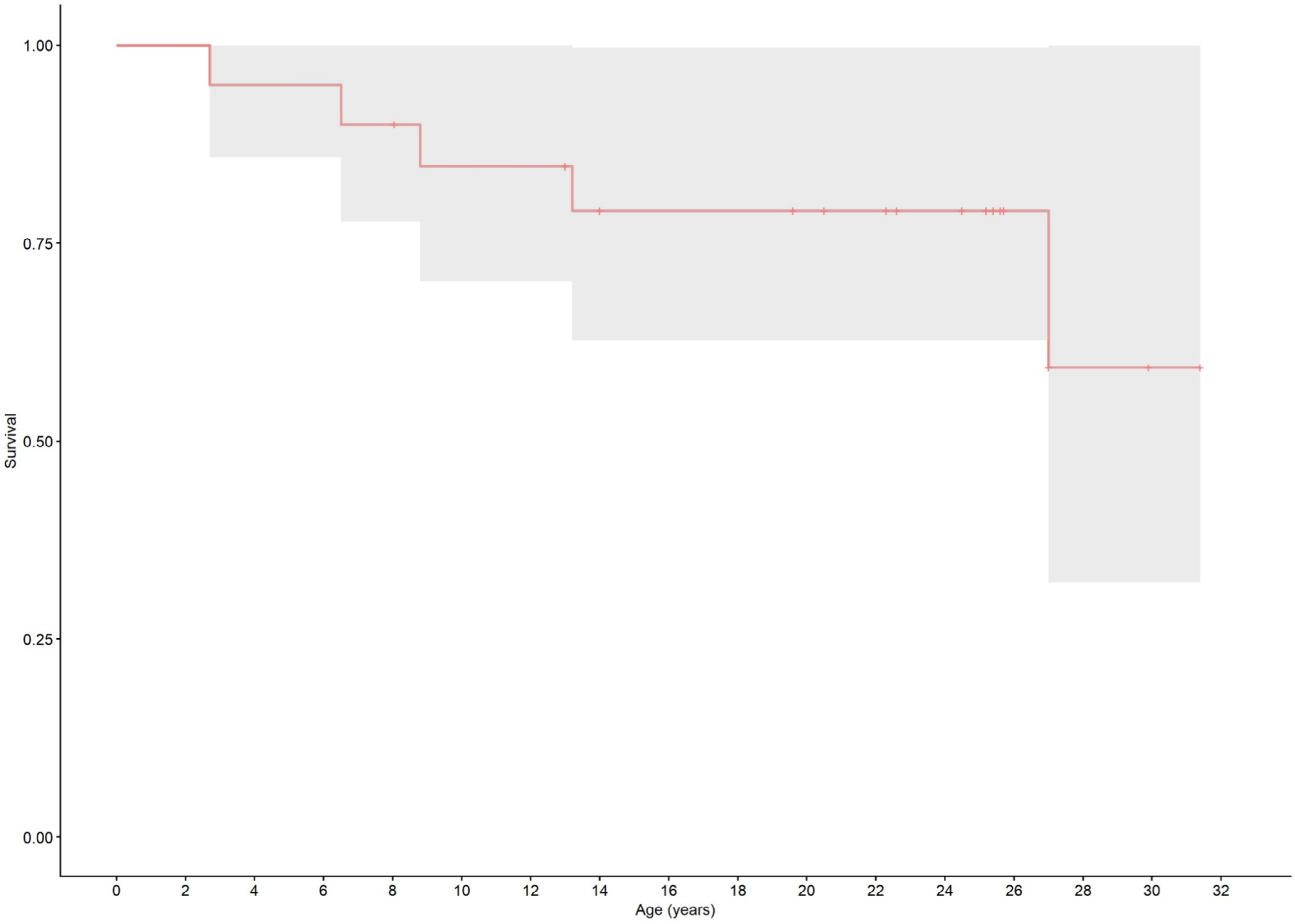
Kaplan–Meier survival curve.

Figure 1 shows the patient Kaplan–Meier survival curve.

Perioperative management included glucose‐lipid infusion and insulin therapy to promote anabolism. Three patients (P1, P3, P5) received perioperative ECMO when indicated by the preoperative aortic clamping test. Postoperatively, protein intake was resumed parenterally from day 2 in patients with evidence of early graft function, and then transitioned to enteral feeding as clinically appropriate. Protein advancement was adjusted rapidly according to clinical and biochemical tolerance within the first two postoperative weeks. Standard immunosuppression consisted of tacrolimus and mycophenolate mofetil, with corticosteroids tapered over the first months post‐transplantation. Exceptions included P6, who received tacrolimus monotherapy, P4, who received ciclosporine, azathioprine, and corticosteroids, and P14 and P20, who received ciclosporine and mycophenolate mofetil. Tacrolimus doses were adjusted according to target trough blood levels.

Organ involvement—including renal, cardiac, neurological, and psychiatric complications—pre‐ and post‐transplantation is summarized in Table 2, together with metabolic parameters. Figure S1 shows paired dot plots for individual data points with median and interquartile range, illustrating pre‐ and post‐transplant plasma and urinary metabolite concentrations.

**TABLE 2:** Organs involvement before (age in years) and after (age in years) transplant.

	P1	P2	P3	P4	P5	P6	P7	P8	P9	P10
Feeding support										
Before	Yes	Yes	Yes	Yes	Yes	Yes	Yes	No	Yes	Yes
After	No	Yes	No	No	No	No	/	No	No	No
Protein intake (g/day and (g/kg/day))										
Before	24 (0.46)	19 (0.31)	27 (0.41)	NA	19 (0.39)	20 (0.64)	17 (1.06)	18 (0.28)	NA	19 (0.3)
After	50 (0.8)	45 (0.85)	60 (0.96)	NA	50 (1.02)	80 (1.45)	/	42 (0.66)	50 (1.22)	50 (0.84)
Mean urinary 3‐OH‐Propionate (mmol/mol of creatinine)										
Before	121	149	76	239	62	60	9	220	NA	175
After	18	15	8	45	52	5	3	11	5	15
Cardiac										
Before	DCM (16)	DCM (5)	DCM + long QTc (19)	DCM (4)	HF + long QTc (14)	DCM (5)	No	DCM (16)	DCM (6)	No
After	Worse with HF and RD until HT	Better then worse	Better then worse	Better	Worse	Normal	Death	Better	Better then worse	RD (0.5)
Neurological										
Before	CI + Epilepsy (8)	CI	Cereb Sd (NA)	No	CI	CI	CI	CI	No	No
After	Stable	ANE (Peri‐LKT)	ANE (1.1)	No	Stable	PRES (Peri‐LT)	/	ANE (Peri‐LT)	No	ANE (0.25)
Neurosensitive										
Before	No	Deafness (15)	Deafness (17) + optic atrophy (18)	No	Strabism (NA)	No	No	Glaucoma (14)	No	No
After	No	Stable	Stable	No	Stable	No	/	Stable	No	No
Psychiatric										
Before	No	No	No	Mood disorder (NA)	Anxiety (13)	No	No	No	No	Intermittently (21)
After	Mood disorder + psychosis (1)	Mood disorder (3)	Sleep disorder (2)	Stable	Worse + psychosis (7)	No	/	No	Anxiety (9)	No
Renal										
Before–mGFR	No–99	Chronic tubulopathy (14)–25	No–107	No–102	No–70	No–80	No–NA	Yes (20)–71	No–NA	Yes (18)–57
After–mGFR	Yes (6)–54	Yes, better–55	No–105	RI until KT (1)–40	Yes (9)–62	Yes (2)–60	/	Yes, stable–80	No—77	Worse–44

	P11	P12	P13	P14	P15	P16	P17	P18	P19	P20
Feeding support										
Before	Yes	Yes	Yes	Yes	Yes	Yes	Yes	Yes	Yes	Yes
After	No	/	No	No	Yes	/	No	No	No	No
Protein intake (g/day and (g/kg/day))										
Before	12 (0.58)	20 (0.38)	25 (0.49)	20 (0.3)	24 (0.54)	11 (0.7)	NA	NA	8 (0.44)	35 (0.5)
After	30 (1.36)	/	45 (1.24)	50 (0.77)	40 (1.01)	/	40 (0.78)	42 (0.76)	40 (1.5)	50 (0.8)
Mean urinary 3‐OH‐Propionate (mmol/mol of creatinine)										
Before	257	139	47	102	61	20	1366	264	NA	128
After	26	7	7	19	15	1	67	NA	NA	13
Cardiac										
Before	No	DCM (12)	DCM + RD (7)	RD (22)	No	No	DCM + Long QTc (16)	Intermittently (NA)	HCM (7)	DCM + long QTc (19)
After	No	Death	Worse + long QTc and RD	Worse	No	Death	Normal	Normal	Better	Better
Neurological										
Before	CI + Leigh (4)	CI	CI	CI + Cereb Sd (NA)	CI + Leigh (10)	CI	CI	CI + Cereb Sd (NA)	Epilepsy (6.8)	CI
After	Stable	/	ANE (0.5)	ANE (0.5)	Stable	/	Stable	Stable	Stable	ANE (0.8)
Neurosensitive										
Before	Deafness (2)	No	No	No	No	No	Optic atrophy (16)	Deafness + diplopy (NA)	NA	No
After	Stable	/	No	No	No	/	Stable	Stable	NA	No
Psychiatric										
Before	Anxiety (4)	Anxiety (16)	ADHD (9)	Anxiety + Autism (18)	No	No	No	Mood disorder (NA)	No	No
After	Stable	/	Stable	Worse + mood disorder + psychosis (1)	No	/	No	Worse + psychosis (NA)	No	No
Renal										
Before–mGFR	No–75	Yes (17)–47	Yes (10)–57	No–70	No–79	No–NA	No–92	No–86	No–NA	Yes (26)–30
After–mGFR	No–76	/	Worse–27	No–70	Yes–40	/	No–70	Yes (6)–44	Tubulopathy (0.5)–NA	Yes, better–54

Abbreviations: ADHD, attention deficit hyperactivity disorder; ANE, acute neurological episodes; Cereb Sd, cerebellar syndrome; CI, cognitive impairment; DCM, dilated CardioMyopathy; HCM, hypertrophic CardioMyopathy; HF, heart failure; HT, heart transplant; LKT, liver‐kidney transplant; LT, liver transplant; NA, not available; PRES, posterior reversible encephalopathy syndrome; RD, rhythm disturbance; RI, renal insufficiency; Tx, transplant.

### 3.1 Metabolic and Dietary Outcomes

No MDEs were observed during follow‐up after transplantation, except for P20, who experienced one episode of acute neurological decompensation with slight lactate elevation 10 months post‐LKT and one episode of hyperammonemia at 136 μmol/L during a viral illness 3 years after LKT; this patient was being treated for chronic myeloid leukemia with Imatinib.

Urinary metabolites varied widely (e.g., pre‐transplant 3‐hydroxypropionate levels from 9 to 1366 mmol/mol creatinine) but mean values decreased significantly after transplantation: 3‐hydroxypropionate from 190 to 19 mmol/mol creatinine (*p* = 0.0003), tiglylglycine from 56 to 6 mmol/mol creatinine (*p* = 0.0002), 2‐methylcitrate from 337 to 193 mmol/mol creatinine (*p* = 0.004), and propionylglycine from 152 to 11 mmol/mol creatinine (*p* = 0.0005). Plasmatic metabolites showed variable results after transplantation: glycine significantly decreased from 1338 to 309 μmol/L (*p* = 1.2 e‐06), but propionylcarnitine non significantly modified from 62 to 47 μmol/L (*p* = 0.12).

Natural protein intake increased significantly after transplantation, both in absolute terms (from 20 to 48 g/day) and relative to body weight (from 0.5 to 1 g/kg/day, *p* = 3.5e‐07). Only two patients still required support for enteral nutrition post‐transplant. Plasma branched‐chain amino acids concentrations rose without reaching statistical significance: valine from 100 to 138 μmol/L (*p* = 0.052), isoleucine from 36 to 50 μmol/L (*p* = 0.063), and leucine from 64 to 84 μmol/L (*p* = 0.081).

Although not systematically evaluated, patients reported subjective improvements in quality of life. Key contributing factors included improved metabolic stability, a marked reduction in hospital admissions, dietary liberalization, discontinuation of enteral feeding, and reduced caregiver anxiety.

### 3.2 Cardiac Outcomes

**FIGURE 2:**
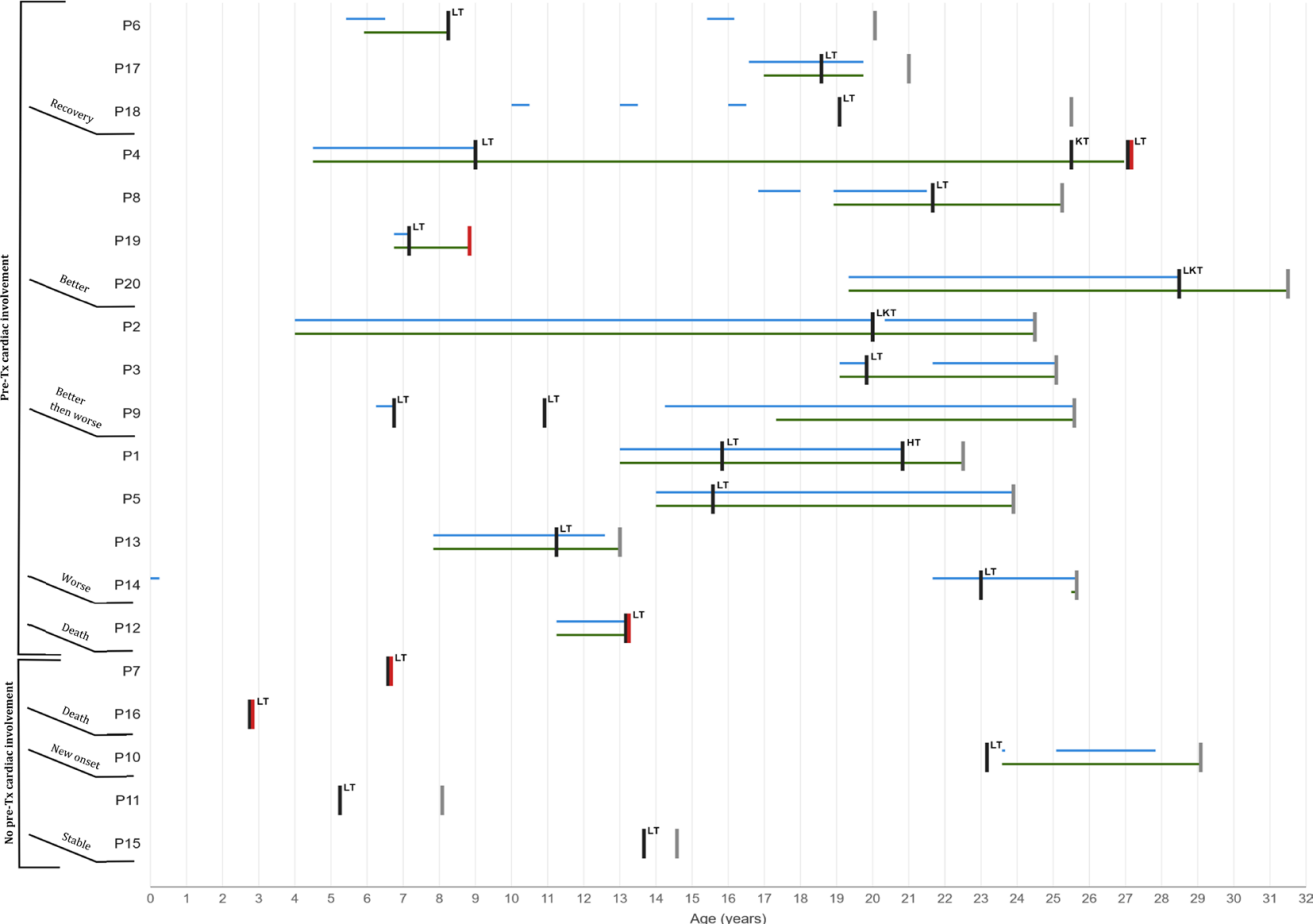
Timeline of cardiac involvement and treatments. Blue line: cardiac disease (DCM or HCM, and/or decreased LVEF and/or rhythm disturbance). Green line: cardiac treatment. Black column: transplant. Grey column: last follow‐up. Red column: death. HT, heart transplant; KT, kidney transplant; LKT, liver kidney transplant; LT, liver transplant; Tx, transplant.

Figure 2 illustrates the timeline of cardiac involvement in all patients, highlighting the variability in the evolution of cardiac symptoms over time.

Cardiac involvement was observed in 75% of patients (*n* = 15) prior to transplant, with a median age of onset of 13 years (range: 2–22 years). Most were dilated cardiomyopathies (*n* = 11), with transient or persistent heart failure. Conduction and rhythm disorders were also documented (*n* = 6), including long QT syndrome, atrioventricular block, or ventricular extrasystole. Among those 15 patients with pre‐transplant involvement, 11 required cardioprotective therapy (mean 2.2 treatments per patient (from 1 to 3)), mainly Angiotensin‐Converting Enzyme (ACE) inhibitors (*n* = 10), beta‐blockers (*n* = 8), and oral ketone bodies (sodium 3‐hydroxybutyrate) (*n* = 7).

Post‐transplant cardiac improvement was observed in 10 patients (67%), including 3 who achieved complete recovery. In most cases, improvement occurred shortly after the procedure, except P17 who recovered within 1 year. Secondary deterioration occurred in 3 patients during follow‐up, from 4 months to 7.5 years. Among patients with pre‐existing cardiac involvement, median left ventricular end‐diastolic diameter (LVEDD) *z*‐score was +0.7 standard deviation (SD) pre‐transplant, +0.8 SD at 1 year, and +1.8 SD at last follow‐up.

One year after transplant, 10 patients were receiving a mean of 2.1 drugs: 8 had been on treatment prior to LT, and one initiated therapy after LT.

At last follow‐up, 10 patients were receiving cardiac therapy (mean 2.5 treatments per patient (ranging from 1 to 4)), including ACE inhibitors (*n* = 6), angiotensin II receptor blockers (*n* = 3), beta‐blockers (*n* = 6), oral ketone bodies (*n* = 4), and sodium‐glucose cotransporter‐2 (SGLT2) inhibitors (*n* = 2).

P1 experienced a progressive worsening of pre‐existing dilated cardiomyopathy after LT, with a nadir LVEF of 10%, a peak LVEDD *z*‐score of +5.4 SD, and onset of arrhythmias; he underwent heart transplantation 5 years after LT with a favorable outcome. P5 also experienced cardiac deterioration, requiring an implantable cardioverter‐defibrillator 7.5 years after LT.

Among the 5 patients without pre‐LT cardiac involvement, two died during the peri‐transplant period from cardiac causes: P7 from myocardial stunning and P16 from arrhythmia. P10 developed mild left ventricular hypertrophy secondary to systemic arterial hypertension and steroids therapy, with rhythm disturbance 2 years after LT. P11 and P15 remained free of cardiac disease, with a short follow‐up (22 months). In patients without pre‐existing cardiac involvement, median LVEDD *z*‐score was stable from −1.2 SD pre‐transplant to −1.1 SD at 1‐year, and −1.1 SD at the last follow‐up; post‐transplant LVEDD data were available for only P10 and P11.

**FIGURE 3:**
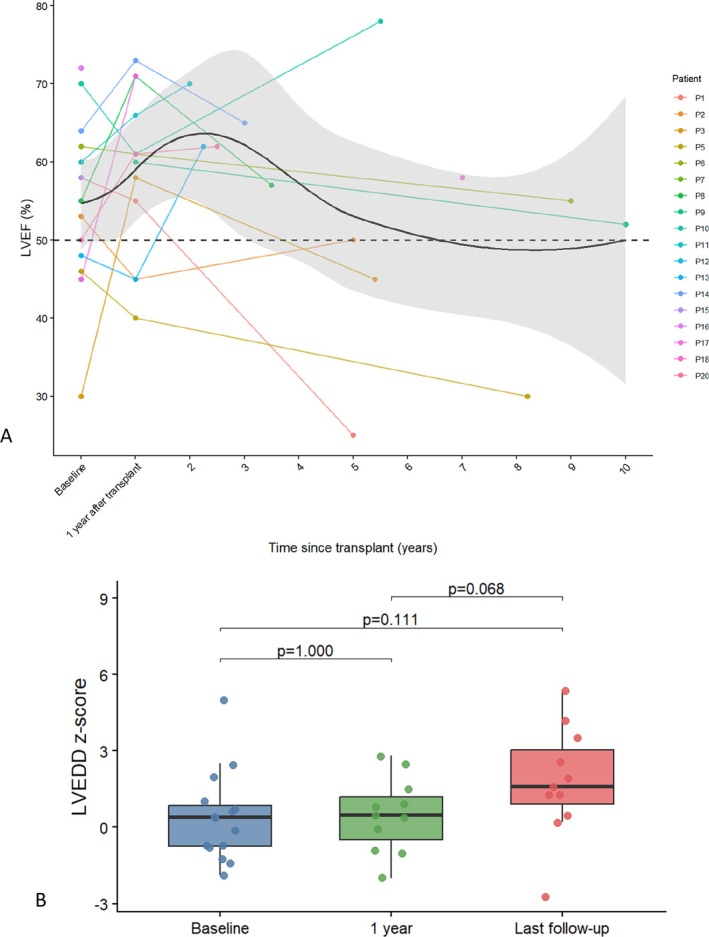
Evolution of ultrasound cardiac parameters before transplantation, at 1‐year, and last follow‐up. (A) (Upper) Evolution of left ventricular ejection fraction (LVEF) by patient. Dashed line: normal range threshold. A locally weighted scatterplot smoothing (LOESS) curve with 95% confidence intervals (shaded area) illustrates the overall trend over time. (B) (Lower) Evolution of left ventricular end‐diastolic diameter (LVEDD) *z*‐score (boxplot). Non‐reported data are not shown.

Figure 3 illustrates the evolution of echocardiographic parameters. Figure 3A displays LVEF prior to transplantation, at 1 year, and at last follow‐up. Although no significant overall change was detected, LVEF tended to increase early during the initial post‐transplant period and then gradually decrease over time.

Figure 3B presents boxplots of LVEDD *z*‐score evolution for all patients. The linear mixed‐effects model demonstrated a significant overall effect of time on LVEDD *z*‐scores (*F*(2, 21.7) = 3.67, *p* = 0.043). Post hoc comparisons did not reveal statistically significant differences after Bonferroni correction, but a trend toward increasing *z*‐scores was observed, suggesting progressive cardiac dilatation.

Among 7 patients transplanted before age 10: P6 achieved full recovery from pre‐existing cardiac dysfunction; P9 initially improved before subsequent deterioration; P11 remained stable without cardiac involvement; and P19 improved but died 1 year later from cirrhosis. In contrast, P7 and P16 died during the peri‐transplant period with cardiac involvement despite no documented pre‐existing cardiac dysfunction.

### 3.3 Neurological Outcomes

Before transplantation, 85% of patients (*n* = 17/20) had chronic neurological symptoms, primarily cognitive impairment, but also cerebellar syndrome, pyramidal signs, and epilepsy, and/or had a history of basal ganglia impairment (Leigh syndrome). Among the 15 patients with cognitive impairment, severity ranged from mild (*n* = 5) to moderate (*n* = 6) and severe (*n* = 4). After transplantation, pre‐existing neurological impairments remained stable in seven patients, while eight patients developed acute neurological events.

Post‐transplant acute neurological episodes can be categorized into distinct patterns: acute symptoms with or without implication of immunosuppressant, with or without MRI abnormalities, and with or without neuropathy.

Five patients developed acute symptoms, improving after immunosuppressant modification, including four with MRI abnormalities and three with neuropathy. P2 developed confusion and visual hallucinations 2 weeks post‐LKT; brain MRI showed bilateral symmetric FLAIR hyperintensities in the striatum, neurological examination and electrophysiological studies were consistent with a chronic moderate distal axonal neuropathy. Switching from tacrolimus to ciclosporin improved neuropathy. P6 developed Posterior Reversible Encephalopathy Syndrome (PRES) syndrome in immediate post‐LT period; MRI showed hyperintensity in the basal ganglia, the posterior fossa, and subcortical regions. Switching from tacrolimus to ciclosporin normalized neurological status. P8 developed confusion and tonic–clonic seizure 2 weeks post‐LT; brain MRI showed symmetric FLAIR hyperintensity in the striatum and slight periaqueductal mesencephalic hyperintensity, with normal spectroscopy. Switching from tacrolimus to ciclosporin improved the neurological status. P10 developed severe sensorimotor axonal neuropathy 3 months after LT; MRI showed hyperintensities in the heads of the caudate nuclei and the lentiform nuclei. Switching from tacrolimus to everolimus improved the neurological status. P14 developed sensory polyneuropathy 6 months after LT. Discontinuation of ciclosporin improved the neurological symptoms.

Symptoms appeared in a catabolic state (peri‐transplant period or gastrointestinal symptoms) for four of them (P2, P6, P8, P10).

Three patients developed acute symptoms without impact of the immunosuppressant, including one with MRI abnormalities and two with neuropathy. P3 developed a right hemiparetic deficit at 13 months post‐LT and subsequently made a complete recovery, without any treatment or immunosuppression modification. MRI was normal and electroencephalogram showed transient slowing in the left temporo‐occipital region. P13 developed distal left sciatic–femoral neuropathy 6 months after LT. Tacrolimus was switched to sirolimus only 1 year later due to decreased kidney function, without any impact on neurological symptoms. P20 developed sensory axonal polyneuropathy with isolated motor involvement of the right tibial nerve 10 months post‐LKT and concomitant slight blood lactate elevation; MRI showed FLAIR and diffusion hyperintensities of the lenticular and caudate nuclei and cerebellar vermis with restricted apparent diffusion coefficient (ADC). P20 was not treated by calcineurin inhibitors (CNI) at this period but by the mechanistic Target Of Rapamycin (mTOR) inhibitor because of primary graft dysfunction of his transplanted kidney, suggesting a metabolic rather than CNI‐related etiology.

Symptoms appeared in a catabolic state (gastrointestinal symptoms) only for P20, who fulfilled criteria for acute Leigh syndrome due to metabolic decompensation.

Beyond acute neurological episodes, other neurological manifestations were recorded. Two patients had treated epilepsy prior to transplantation (P1, P19), and two patients had experienced febrile seizures on two occasions each (P11, P16). None of these patients experienced recurrence after transplantation. No abnormal movements were documented in any patient, either before or after transplantation.

Only three patients (P4, P9, P10) had no neurological involvement prior to transplantation. P4 and P9 remained free of neurological disease after transplantation, both having undergone early transplantation at ages 9 and 6 years respectively. P10, despite no pre‐transplant neurological involvement, developed post‐transplant neurological complications as described above.

Sensorineural impairments were documented in 37% of patients (*n* = 7/19) prior to transplantation, including five with ophthalmological abnormalities (two with optic atrophy, one with glaucoma, two with diplopia) and four with varying degrees of hearing loss. These symptoms typically manifested at a median age of 15.5 years (range 2–18 years). After transplantation, these impairments remained stable.

### 3.4 Psychiatric Outcomes

Psychiatric symptoms were documented in 40% of patients (*n* = 8) before transplantation, with a median age at onset of 14.5 years (range 4–21 years). Symptoms worsened in three patients after LT (P5, P14, P18) and four patients developed new‐onset psychiatric symptoms after transplantation (P1, P2, P3, P9). P3 developed a sleep disorder characterized by nocturnal terrors and other parasomnias. Two patients (P1, P18) required multiple psychiatric hospitalizations for psychotic symptoms. These deteriorations occurred at a median of 2.5 years post‐transplant (median age 20.9 years). Only one patient (P10) showed marked improvement in psychiatric status after LT.

At last follow‐up, 59% of patients (*n* = 10) exhibited psychiatric manifestations; 70% of those (*n* = 7) required therapeutic management. Reported symptoms included anxiety disorders (*n* = 4, 24%), mood disorders (*n* = 5, 29%), and psychotic symptoms (*n* = 4, 24%).

Among patients transplanted before the age 10: P4 remained stable; P6 and P11 remained free of psychiatric symptoms; P19 was free of psychiatric symptoms in the year before his death. P9 developed new psychiatric symptoms after transplantation.

### 3.5 Renal Outcomes

Chronic kidney disease (CKD) increased from 30% of patients (*n* = 6) pre‐transplant (mean mGFR 72 mL/min/1.73 m^2^) to 70% (*n* = 12) post‐LT (mean mGFR 60 mL/min/1.73 m^2^). Two patients underwent initial combined LKT because of reduced mGFR (P2: 25 mL/min/1.73 m^2^; P20: 30 mL/min/1.73 m^2^). Another patient (P4) required KT 16 years after LT for progressive renal failure (40 mL/min/1.73 m^2^). Initial post‐LT immunosuppression included a nephrotoxic CNI (ciclosporin or tacrolimus) in all patients. CNI was subsequently switched to an mTOR inhibitor for nephroprotection in P1 and P13, and for primary kidney graft dysfunction in P20, resulting in transient GFR improvement in P1 and P13, and sustained improvement in P20.

Among patients transplanted early, only two (P9 and P11) had normal renal function at last follow‐up.

## 4 Discussion

Our cohort is among the largest reported series of PA patients who underwent liver transplantation (LT), spanning 33 years [4] with individual follow‐up from 1 to nearly 20 years. Published LT experience in organic acidemias is predominantly weighted toward methylmalonic acidemia (MMA) [7, 11, 16] while PA‐specific data remain limited [17, 18]. LT is a well‐established treatment for selected metabolic disorders and inborn errors of metabolism represents the second most common indication for pediatric LT [5, 19, 20, 21]. It can be performed either for direct liver involvement (usually not the case in PA) or as a form of “enzyme replacement therapy” to correct systemic metabolic enzyme defect: the latter indication has been increasingly applied in organic acidemias with chronic metabolic instability [1, 2, 5]. Despite optimal management according to current standards of care, long‐term prognosis in PA remains poor, characterized by multisystemic complications and early mortality [3, 22, 23]. Hepatic involvement in PA, although uncommon, has been previously described in organic acidemias and may represent an underrecognized complication of chronic metabolic disease [24]. In our cohort, one patient underwent LT specifically for biliary cirrhosis with portal hypertension attributed to PA, illustrating the potential for direct hepatic injury independent of acute metabolic decompensation.

Post‐LT dietary management allowed a partial but significant relaxation of protein restriction [8]. Based on our experience with MMA, our target post‐LT protein intake is approximately 1 g/kg/day to prevent nutritional deficiency and limit renal risk without full protein liberalization; this target was reached despite no clear improvement in plasma branched‐chain amino‐acids levels. The need for nutritional support decreased substantially—only two patients required ongoing nutritional support post‐LT (one recently transplanted).

LT improves metabolic stability and reduces the frequency of MDEs [7, 17]. In our cohort, we observed no post‐LT episodes of metabolic acidosis but one episode of hyperammonemia, consistent with prior reports [18, 25]. Biochemically, urinary 3‐hydroxypropionate and other metabolites fell after LT; however, propionic acidemia lacks robust, reproducible biomarkers for routine monitoring because urinary metabolites are highly variable. Although mean post‐LT values decreased, urinary 3‐hydroxypropionate serves primarily as a diagnostic marker rather than a reliable monitoring tool [26]. More sensitive methods, such as plasma 3‐hydroxypropionate quantification [27] could enhance clinical monitoring, but these assays are not yet available in France. Secondary metabolic markers, such as plasma glycine and urinary 2‐methylcitrate, are less routinely monitored in our clinical practice; nonetheless, both showed a statistically significant decrease following transplantation. Cerebrospinal fluid levels of these metabolites were not measured in our cohort; however, studies in MMA have reported no significant change in CSF metabolite concentrations following liver transplantation, suggesting that hepatic enzyme replacement does not fully normalize the neurochemical environment: a finding potentially relevant to PA as well [26, 28].

Although not measured with systematic assessment, dietary relaxation and reduced MDE frequency translated into meaningful improvement in quality of life for many patients, supporting LT as a therapeutic option in PA, findings concordant with prior published series [29, 30, 31, 32, 33, 34, 35, 36]. Recent studies have highlighted the use of both disease‐specific (MetabQoL) and generic (PedsQL) quality‐of‐life instruments to document post‐transplant improvements [36, 37]. However, systematic quality‐of‐life assessment using validated instruments was only recently implemented at our center, and quantitative data was not available for most patients in this cohort.

A key observation is the limited and inconsistent effect of LT on chronic organ damage, notably cardiac disease. Earlier small series—including our own—suggested possible reversibility of PA‐related cardiomyopathy after LT [6], although follow‐up was often short. Based on these preliminary findings, cardiac involvement was considered one of the potential indications for LT [1], notably by our team. However, in our cohort, cardiac outcomes were highly variable: several patients developed secondary deterioration or new‐onset cardiac dysfunction after LT. This deterioration may be severe enough to require secondary heart transplantation, as observed in P1 [18]. Additionally, many peri‐operative deaths occurred in patients with cardiac involvement, even without pre‐existing documented cardiomyopathy. These events often coincided with high systemic and myocardial oxygen demand driven by medical stress, systemic inflammation, or sepsis, suggesting cardio‐metabolic uncoupling as a possible mechanism. Although a time effect on LVEDD *z*‐scores reached nominal significance (*p* = 0.043), lack of post hoc significance likely reflects small sample size and limited power.

Some published series have reported cardiac improvement following LT, including reversal of cardiomyopathy, though these observations are often based on small cohorts with limited follow‐up [6, 11, 29, 38, 39, 40, 41, 42]. In contrast, recurrence or new‐onset cardiac dysfunction post‐LT—occasionally leading to cardiac‐related mortality—has been increasingly recognized [9, 10, 43, 44, 45] consistent with the variability in our findings [9, 30, 46]. We found no clear impact of age at LT on cardiac trajectories (no difference in LVEDD or LVEF trends for those transplanted before vs. after age 10).

Cardiac complications in PA exhibit wide variability in age of onset and severity, predominantly presenting as dilated cardiomyopathy and arrhythmias, such as prolonged QTc intervals [47, 48, 49, 50, 51, 52, 53, 54]. The multifactorial pathophysiology underlying cardiac disease in PA cannot be solely attributed to chronic intoxication, which could be improved or prevented by LT [6, 51, 52, 55, 56, 57]. These observations highlight the complexity and unpredictable nature of cardiac outcomes, suggesting that cardiomyopathy should not be considered a routine indication for LT, given the inconsistent efficacy of transplantation in preventing or managing cardiac disease. In contrast, we are unable to provide insights into the potential benefits of earlier transplants, as our cohort does not include enough patients transplanted at a younger age.

We recorded severe acute neurological events, including MRI abnormalities, neuropathy, and Leigh syndrome, in eight patients. Others have reported similar acute neurological symptoms post‐LT, often occurring without identifiable triggers or well‐understood pathophysiological mechanisms [33, 34, 43]. In our cases most acute episodes occurred during catabolic state; but some events lacked a clear trigger. Analogous complications are more commonly documented in MMA, where secondary mitochondrial dysfunction—potentially exacerbated by CNIs, especially tacrolimus—has been proposed [28, 58, 59, 60]. Clinical differentiation between CNI‐related neurotoxicity and metabolic decompensation remains challenging. Martinelli et al. [28] proposed radiological, biological, and clinical criteria to aid this distinction, partly based on plasma methylmalonic acid levels measured during acute episodes in MMA patients. However, no equivalent reliable biomarker has been identified in PA, limiting the applicability of this framework to our cohort.

Interestingly, while most neurological episodes in our series improved following immunosuppressant modification—suggesting a significant contribution of CNI toxicity—we observed more frequent MRI abnormalities than those described by Martinelli et al. [28] in CNI‐induced events. Furthermore, one patient (P20) experienced acute neurological decompensation in the complete absence of CNI therapy, demonstrating that metabolic mechanisms can operate independently of immunosuppressive toxicity. It is also possible that persistent accumulation of toxic metabolites in the cerebrospinal fluid, despite improved plasma levels, contributes to neurological injury [28]. Taken together, these observations suggest that LT does not fully correct secondary energetic dysfunction or the full spectrum of pathophysiological mechanisms driving neurological manifestations in PA [61, 62]. In highly catabolic situations, aggressive promotion of anabolism (emergency dextrose/protein‐sparing strategies) and cautious use of CNIs are reasonable management principles.

Neurodevelopmental outcomes following LT are increasingly well‐characterized, with a general trend towards stabilization or even improvement in developmental quotients and clinical symptoms [29, 34, 35, 63]. Improvements in brain MRI were also reported [28, 64]. However, some studies suggest that patients transplanted for metabolic disorders may exhibit poorer cognitive outcomes compared to patients with other indications [65]. In our series, LT did not reverse existing neurological deficits but generally contributed to clinical stabilization. Notably, two of three patients without neurological impairment at last follow‐up had been transplanted before the age of 10. While we found no correlation between age at transplantation and occurrence of acute neurological complications or long‐term neurocognitive outcome, early transplantation, especially before the onset of neurological signs, may be associated with better neurological preservation. This hypothesis is supported by the recent study by Bo et al. [18] which reported improvement in developmental delay in the majority of their cohort, transplanted at a notably younger median age of 1.4 years.

Psychiatric disorders emerged commonly in adolescence in our cohort and were sometimes severe, impairing autonomy and quality of life; several patients required psychotropic medication and occasionally hospitalization. Autism spectrum features have been reported in PA [66, 67], with hypotheses implicating propionate neurotoxicity [68, 69, 70]. Other psychiatric manifestations have also been described [71, 72, 73], notably in natural history cohorts where self‐reported psychological disturbances appeared more frequent than in the general population [74]. Psychiatric complications following liver transplantation have likewise been reported in the general transplant population, affecting approximately 15% of recipients regardless of the underlying diagnosis [75]. The considerably higher frequency of 60% observed in our cohort probably reflects multiple contributing factors: persistent accumulation of toxic metabolites in the cerebrospinal fluid despite plasma improvement [28], inherent adolescent neurodevelopmental vulnerability, the potential added impact of CNI‐related neurotoxicity [76, 77], and the natural history of PA itself. In our data, psychiatric trajectories appeared largely independent of age at LT and could progress despite successful transplantation, underscoring the need for regular psychiatric monitoring and a high index of clinical suspicion in this population.

Chronic kidney disease was common before LT and often progressed after transplantation. Kidney complications in PA remain poorly characterized [31, 78]. Quintero et al. [31] reported only two cases of moderate renal dysfunction following liver transplantation, both occurring in patients without pre‐existing renal impairment. Currently, no consensus exists regarding a GFR threshold for the indication of combined kidney–liver transplantation; at our center we consider combined transplantation when GFR falls below 50–60 mL/min/1.73 m^2^, balancing organ availability and the immunological benefit of combined liver and kidney transplantation from the same donor. The mean GFR declined by ~10 mL/min/1.73 m^2^ in our cohort post‐LT. The decline is likely multifactorial, reflecting the cumulative nephrotoxic burden of CNI, the natural progression of PA‐related renal disease, and potentially hemodynamic factors related to the transplant procedure itself. Transition to mTOR inhibitors or mycophenolate mofetil may attenuate further decline, though longer follow‐up is required to confirm this.

Overall crude survival in our cohort was 75% at last follow‐up, broadly comparable to previously reported survival rates in transplanted PA patients [79]. Some series report similar outcomes, including approximately 80% survival at a median follow‐up of 2.2 years [7, 17] but 70% survival at 20 years [80]; somewhat higher than our Kaplan–Meier estimate of approximately 50% at 20 years, though this figure should be interpreted with caution given the small number of patients with sufficient follow‐up duration to contribute to this estimate and the historical nature of our earliest transplantation. However, more recent cohorts—including a European series with a median transplantation age of 2.7 years [7, 17] and a recent Chinese cohort reporting a median of 1.4 years [18]—report substantially higher survival rates exceeding 90% [5, 18], suggesting that evolving patient selection, earlier transplantation, and improved perioperative management may collectively contribute to better outcomes. Whether earlier transplantation independently drives this survival advantage remains difficult to establish. On one hand, transplantation before the onset of organ damage is an appealing strategy, as argued in MMA [7, 35] and is supported by the better neurodevelopmental outcomes reported in younger transplanted cohorts [18]. On the other hand, peri‐transplant mortality has been reported to be higher in children under 3 years of age [7], and our own data illustrate this tension: among seven patients transplanted before age 10, three died, two of whom in the peri‐transplant period. Crucially, peri‐operative cardiac mortality was not confined to patients with pre‐existing cardiomyopathy—two patients without documented cardiac involvement prior to LT died of cardiac causes in the peri‐transplant period—underscoring the inherent cardiac vulnerability of PA patients regardless of baseline cardiac status [9, 10]. This observation highlights that cardiac risk in PA is not fully predicted by pre‐transplant cardiac evaluation, and that individualized cardio‐circulatory management, including prophylactic or rescue ECMO support within a framework of closely monitored and continuously adapted energy provision, constitutes one of the key perioperative challenges in this setting.

Several limitations of this study warrant consideration. The retrospective design and the extended study period (spanning over three decades) introduced substantial heterogeneity because surgical, anesthetic, and peri‐operative metabolic management have evolved substantially [7, 31, 79, 81, 82, 83, 84]. Diagnostic and management practices also changed over time, and patients were transplanted at a wide range of ages (median 13.6 years), further limiting comparability within the cohort and with younger series. Current management at our center follows a standardized perioperative protocol encompassing pre‐ and post‐transplant metabolic optimization, nutritional support, and cardiorespiratory monitoring; long‐term follow‐up now incorporates systematic organ function assessments and validated quality‐of‐life questionnaires. However, such protocolized approaches were not consistently applied throughout the entire study period, particularly for patients transplanted in the early years of the cohort, which may have resulted in underreporting of complications and limited the systematic collection of patient‐reported outcomes.

The management of PA and related intoxication‐type metabolic disorders is undergoing significant evolution, particularly with the advent of novel therapeutic approaches [85, 86, 87]. Among these, the development of mRNA‐based therapies may substantially improve clinical outcomes, either as a long‐term disease‐modifying strategy or as a bridge‐to‐transplant approach aimed at reducing pre‐transplant organ damage.

## 5 Conclusion

Liver transplantation should be considered on a case‐by‐case basis for patients with severe propionic acidemia. The procedure is challenging and associated with substantial morbidity and mortality. LT clearly improves metabolic stability and dietary management, but these benefits must be balanced against persistent risks—notably cardiac disease, psychiatric comorbidities, and acute neurological complications. Earlier transplantation, before comorbidity onset, could be beneficial, but we were not able to demonstrate a correlation between age at LT and prevention of organ involvement. Cardiomyopathy alone should not constitute a systematic indication for transplantation: while transient improvements may be observed, they are inconsistent, often not sustained, and unlikely to prevent further deterioration. Robust emergency protocols to promote anabolism during catabolic episodes remain essential to reduce new neurological events.

Emerging therapies, including mRNA‐based approaches, may offer promising clinical outcomes and potentially reduce morbidity and mortality associated with both PA and transplantation procedures.

## Author Contributions

Project design: A.B., C.‐M.B., T.M., P.D.L., and F.L. Data collection: T.M. and C.‐M.B. Redaction: T.M., C.‐M.B., M.S., P.D.L., and A.B. All co‐authors critically reviewed the study.

## Funding

The authors have nothing to report.

## Consent

In accordance with French legislation, patients or their legal representatives were informed during routine consultations that their medical data might be used for research purposes; lack of opposition was systematically recorded. All data were anonymized and stored securely in a password‐protected database, complying with French and European data protection regulations.

## Conflicts of Interest

Payment or honoraria for lectures, presentations, speakers bureaus, manuscript writing or educational events, by IECure, Ultragenyx, Immedica, Travere, to Pr Manuel Schiff. The other authors declare no conflicts of interest.

## Supporting information


**Figure S1:** Biochemical parameters before and after liver transplantation. (A–E) Plasma concentrations of isoleucine, valine, leucine, glycine, and propionylcarnitine (μmol/L). (F–I) Urinary excretion of 2‐methylcitric acid, propionylglycine, tiglylglycine, and 3‐hydroxypropionic acid (mmol/mol creatinine). Each dot represents an individual patient. Horizontal bars indicate the median and interquartile range (IQR). The grey shaded area represents the IQR of the post‐transplantation group. Only patients with complete paired measurements were included in statistical analyses. Normality of differences was assessed by the Shapiro–Wilk test; paired *t*‐test or Wilcoxon signed‐rank test was applied accordingly. *p < 0.01; **p < 0.001; ***p < 0.0001; n.s., not significant.

## Data Availability

Data supporting the results reported in the article are available on demand to the corresponding author.
