# Supplementary material for: Long Term Follow‐Up After Transplantation in Propionic Acidemia: A Retrospective French Pediatric and Adult Cohort Study
Source: J Inherit Metab Dis. 2026 Jun 25;49(4):e70216. doi: 10.1002/jimd.70216 (PMC13297872; doi:10.1002/jimd.70216)
Supplement: Supplementary file 1 — Figure S1: Biochemical parameters before and after liver transplantation. (A–E) Plasma concentrations of isoleucine, valine, leucine, glycine, and propionylcarnitine (μmol/L). (F–I) Urinary excretion of 2‐methylcitric acid, propionylglycine, tiglylglycine, and 3‐hydroxypropionic acid (mmol/mol creatinine). Each dot represents an individual patient. Horizontal bars indicate the median and interquartile range (IQR). The grey shaded area represents the IQR of the post‐transplantation group. Only patients with complete paired measurements were included in statistical analyses. Normality of differences was assessed by the Shapiro–Wilk test; paired t‐test or Wilcoxon signed‐rank test was applied accordingly. *p < 0.01; **p < 0.001; ***p < 0.0001; n.s., not significant. [file JIMD-49-0-s001.docx]

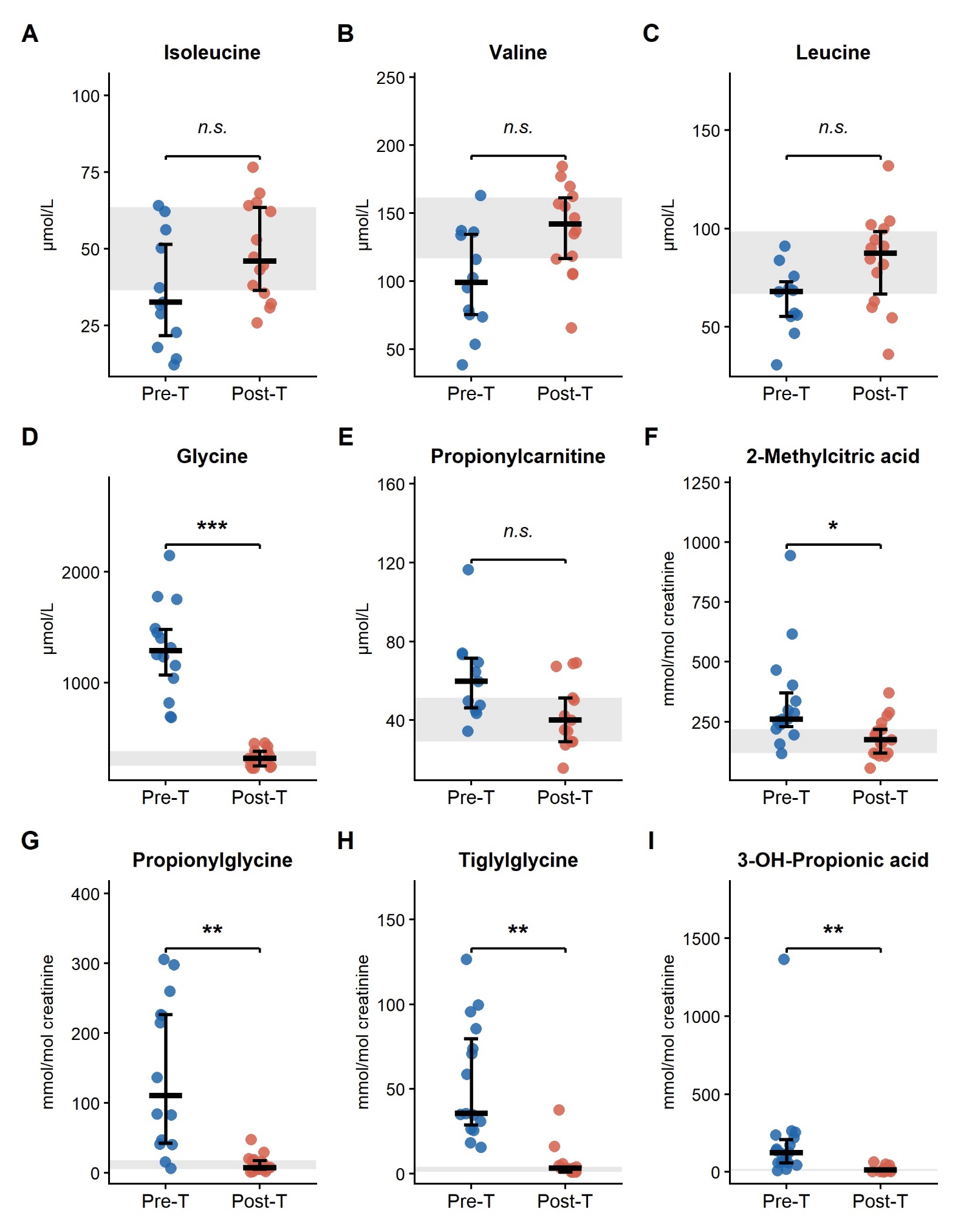


**Supplementary Figure 1.** **Biochemical parameters before and after liver transplantation.**

A–E, Plasma concentrations of isoleucine, valine, leucine, glycine, and propionylcarnitine (µmol/L). F–I, Urinary excretion of 2-methylcitric acid, propionylglycine, tiglylglycine, and 3-hydroxypropionic acid (mmol/mol creatinine). Each dot represents an individual patient. Horizontal bars indicate the median and interquartile range (IQR). The grey shaded area represents the IQR of the post-transplantation group. Only patients with complete paired measurements were included in statistical analyses. Normality of differences was assessed by the Shapiro-Wilk test; paired t-test or Wilcoxon signed-rank test was applied accordingly. *p < 0.01; **p < 0.001; ***p < 0.0001; n.s., not significant.
